# Sexual Dimorphic Responses in Lymphocytes of Healthy Individuals after *Carica papaya* Consumption

**DOI:** 10.3389/fimmu.2017.00680

**Published:** 2017-06-09

**Authors:** Nur Ramziahrazanah Jumat, Mun Yee Chong, Zainina Seman, Rosita Jamaluddin, Nyet Kui Wong, Maha Abdullah

**Affiliations:** ^1^Immunology Unit, Department of Pathology, Universiti Putra Malaysia, Serdang, Malaysia; ^2^Hematology Unit, Department of Pathology, Universiti Putra Malaysia, Serdang, Malaysia; ^3^Department of Dietetics and Nutrition, Faculty of Medicine and Health Sciences, Universiti Putra Malaysia, Serdang, Malaysia; ^4^Biotechnology Program, Faculty of Science and Natural Resources, Universiti Malaysia Sabah, Kota Kinabalu, Malaysia

**Keywords:** natural killer cells, T cells, CD25, CD69, CD107a, sex hormones, *Carica papaya*

## Abstract

Sexual dimorphism in immune response is widely recognized, but few human studies have observed this distinction. Food with endo-immunomodulatory potential may reveal novel sex-biased *in vivo* interactions. Immunomodulatory effects of *Carica papaya* were compared between healthy male and female individuals. Volunteers were given fixed meals supplemented with papaya for 2 days. Changes in blood immune profiles and hormone levels were determined. In females, total natural killer (NK) cell percentages decreased (12.7 ± 4.4 vs 14.6 ± 5.8%, *p* = 0.018, *n* = 18) while B cells increased (15.2 ± 5.5 vs 14.5 ± 5.0, *p* = 0.037, *n* = 18) after papaya consumption. Increased 17β-estradiol (511.1 ± 579.7 vs 282.7 ± 165.0 pmol/l, *p* = 0.036, *n* = 9) observed in females may be crucial to this change. Differentiation markers (CD45RA, CD69, CD25) analyzed on lymphocytes showed naïve (CD45RA^+^) non-CD4^+^ lymphocytes were reduced in females (40.7 ± 8.1 vs 46.8 ± 5.4%, *p* = 0.012, *n* = 8) but not males. A general suppressive effect of papaya on CD69^+^ cells, and higher percentage of CD69^+^ populations in females and non-CD4 lymphocytes, may be relevant. CD107a^+^ NK cells were significantly increased in males (16.8 ± 7.0 vs 14.7 ± 4.8, *p* = 0.038, *n* = 9) but not females. Effect in females may be disrupted by the action of progesterone, which was significantly correlated with this population (*R* = 0.771, *p* = 0.025, *n* = 8) after papaya consumption. In males, total T helper cells were increased (33.4 ± 6.4 vs 32.4 ± 6.1%, *p* = 0.040, *n* = 15). Strong significant negative correlation between testosterone and CD25^+^CD4^+^ lymphocytes, may play a role in the lower total CD4^+^ T cells reported in males. Thus, dissimilar immune profiles were elicited in the sexes after papaya consumption and may have sex hormone influence.

## Introduction

Sexual dimorphism in immune response of the innate and adaptive systems has been extensively reviewed in literature and manifested in differential resistance to infections. Females in general are better than males in defense against a variety of bacterial, viral, and parasitic infestations (1, 2). Dimorphism in severity and pathogenesis are also apparent as certain infectious and parasitic diseases increase mortality in females but not males (3). Sex hormones and sex chromosome-related genes such as toll-like receptors, cytokine receptors involved in T-cell and B-cell activity in the X chromosome and inflammatory pathway genes in the Y chromosome (4, 5) are expected to be major contributors to this disparity.

Sex differences in non-communicable diseases are also observed in particular autoimmune diseases (6) as well as metabolic diseases, hypertension, cardiovascular diseases, psychiatric, neurological disorders, and cancer (7). More than 80% of all patients with autoimmune disease are women. Sex as a risk factor in coronary artery disease is observed in incidence rate and also age of onset, progression, treatment efficacy, morbidity, and mortality (8). These dissimilarities are attributed to genetic as well as hormonal differences and interactions and responses to environmental factors including infection, diet, drugs, stress, as well as behavior. Host hormone interactions with commensal gut microbiome are suggested to shape the microbiome composition (9), which is essential in immune homeostasis. Thus, sex matters and must be a consideration when decisions around therapeutic intervention strategies are being developed (9). Substantial data have accumulated from many epidemiological studies. *In vitro* studies demonstrating effects of sex hormones on immune cell subsets are well documented. However, *in vivo* human studies are still lacking.

Immunomodulatory potentials of phytochemicals and purified components of natural products are well studied. Whole food and its nutrients also have immunomodulatory effects, health healing potential, and play a role in homeostatic maintenance of the immune system but are less investigated. Grape juice consumption mobilized gamma–delta T cells and maintained immunity in healthy humans (10). A study on mice showed ginseng berry extract injected into mice exerted immunostimulatory effect by increasing pro-inflammatory molecules in dendritic cells from spleen after 24 h treatment (11). In diseased models, polysacccharide fractions from *Momorica charantia*, an edible medicinal vegetable, significantly increased various immune indexes to normal control levels in cyclophosphamide-induce immunosuppressed mice (12). Feijoa sellowiana Berg var. coolidge fruit juice consumption was shown to have anti-inflammatory activity on edema-induced mice within first hour of treatment (13) while agipenin, a natural flavonoid reduced neuroinflammation by protection against damage from dendritic cells stimulated T cells in experimental autoimmune encephlalomyelitis mouse models (14). Dietary polyphenols were found to exert a regulatory role on dendritic cell function. Researches in human are few and still new but represent an area of scientific need, opportunity, and challenge (15).

*Carica papaya* fruit is commonly consumed worldwide. It has high antioxidant activity (16) and rich in phytochemicals such as flavonoids (17). Different plant parts such as fruit, leaf, seed, root, bark, and flowers have been used as health treatments in tropical countries where it is grown. The seeds of papaya, however, have contraceptive effect on male fertility as well as manifest antifertility, anti-implantation, and abortifacient activity in female rats (18) suggesting a possibility to alter sex hormone levels.

We examined the potential of papaya fruit to modulate immune profiles and sex hormones in healthy male and female individuals. We observed differential immune profiles in sexes after papaya consumption, which may be influenced by sex hormones.

## Methodology

### Subjects

Apparently healthy individuals, age 18–35 years old, with no history of chronic or acute illness, no recent history of vaccination, piercing or blood transfusion, and not on medication or supplements were included. A total of 33 subjects, 15 males and 18 females, were recruited and underwent a papaya supplementation experiment. Subsequent lab investigations, however, were not conducted on all samples collected. Female subjects were enlisted during their second or third week after onset of menstruation and determined not on oral contraceptive. This study was approved by the Medical Research Ethics Committee, Faculty of Medicine and Health Sciences, Universiti Putra Malaysia. All procedures complied with the principles of the Declaration of Helsinki. Informed written consents were obtained from participants.

### Papaya Supplementation Experimental Design

A 5-day experiment was designed. Two subjects, one male and one female, were randomly selected at a time. Food intake was controlled with provision of standard meals consisting of bread/rice/noodle, chicken, vegetables, and liquid. The menu for day 3 and day 4 were replicates of day 1 and day 2, respectively. A pre-exposure period of 2 days (without papaya) was followed by 2 days with 100 g of fresh papaya fruit (fruit color index 4) in the day’s three major meals. Daily dietary recall was conducted to confirm that the fruit provided each time was completely consumed while a medical call was carried out to determine no adverse effects. A peripheral blood sample (20 ml) was collected in K_2_EDTA vacutainers in the morning before meal of day 3 (0 h) and day 5 (48 h). Either whole blood or peripheral blood mononuclear cells (PBMCs) was used in the experiments. Whole blood was used directly after withdrawal. PBMC was isolated by density centrifugation with Ficoll-Paque (GE Healthcare, USA) and stored over liquid nitrogen until further use. Plasma was collected and stored at −80°C for measurement of sex hormones levels.

### Lymphocyte Subset Enumeration

Percentages and absolute counts of lymphocyte subsets in whole blood were determined with the BD Multitest™ IMK kit (BD Biosciences, USA) containing antibodies against CD45, CD3, CD4, CD8, and CD16^+^CD56 together with BD Trucount tubes, according to procedures provided by manufacturer. All samples were tested. Cells were acquired on a BD FACSCanto flow cytometer (BD) and analyzed with FACSCanto Clinical Software (BD).

### Expression of CD45RA, CD69, and CD25 on CD4^+^ T Cells and Non-CD4^+^ Lymphocytes

From here on, only nine paired samples from males and nine paired samples from females were tested. Subsequent missing samples were due to loss of data during a transition period. For surface marker studies, heparinized whole blood sample (100 μl) was incubated with monoclonal antibodies to CD4-PerCP, CD45RA-FITC, CD25-APC, and CD69-PE purchased from Becton Dickinson (USA), following standard procedures. Briefly, after 20 min incubation in dark at 4°C, red blood cells were lysed with 1× lysing solution (Becton Dickinson, USA). After washing with 1× PBS, cells were re-suspended in 500 μl of 2% paraformaldehyde. Ten thousand events gated on CD4^+^ bright population were acquired on a flow cytometer (BD LSR-Fortessa) and analyzed using FACSDiva (Becton Dickinson, USA).

### Surface Marker Expression of Interleukin (IL) Receptors (IL-12Rβ2, IL-15Rα, IL-21R) on CD8^+^ T and NK Cells

Whole blood (600 μl) diluted with equal volume of RPMI 1640 medium without FBS was dispensed in BD Falcon polystrene tubes and incubated with 400 ng/ml phorbol myristate acetate (PMA) (Sigma-Aldrich, USA) together with calcium ionophore (Sigma-Aldrich, USA) and golgi stop containing monensin (Becton Dickinson, USA) for 6 h at 37°C and 5% CO_2_. After incubation, four-color staining (FITC/PE/APC/PerCP) for lineage markers, CD3, CD8, and CD56 and one of the surface IL receptor, IL-12Rβ2-PE, IL-15Rα-PerCP, or IL-21R-PE was performed. Subsequently, RBC was lysed with 1× lysing solution (Becton Dickinson, USA) following manufacturer’s protocol and then fixed with 2% paraformaldehyde before analysis using BD FACSDiva software on LSR-Fortessa flow cytometer (BD).

### Intracellular Cytokine Staining for Interferon-γ (IFN-γ)

The same stimulation procedure as above (cytokine receptors) was carried out. After 6 h incubation, cell surface staining for lineage specific markers (CD3, CD8, CD56) was performed. To detect IFN-γ secretion, cells were fixed with 2% paraformaldehyde followed by permeabilization with BD Perm/Wash solution before staining for intracellular IFN-γ PE-labeled antibody. Cells were analyzed on BD LSR-Fortessa flow cytometer (BD).

### CD107a Degranulation Assay on CD8^+^ T and NK Cells

Peripheral blood mononuclear cell (1 × 10^6^ cells/ml) from volunteers were re-suspended in 500 μl of complete RPMI 1640 medium in BD Falcon polystrene tubes and incubated with 100 ng/ml PMA with calcium ionophore and golgi stop-containing monensin. PBMC was also incubated with monoclonal antibody to CD107a. Tubes were vortexed gently and incubated for 5 h in dark at 37°C with 5% CO_2_. Subsequently, cells were washed with PBS, stained with monoclonal antibodies specific for CD3, CD8, and CD56 and analyzed on BD LSR-Fortessa flow cytometer (BD, USA).

### Sex Hormone Assay

Measurement of sex hormone levels was outsourced to a local pathology laboratory for detection of 17β-estradiol, progesterone, and testosterone serum levels using System ARCHITECT ci8200 together with respective kits. Normal ranges were provided with the kits. Levels of sex hormones (17β-estradiol, progesterone, and testosterone) were then correlated with immune profiles determined in the study.

### Statistical Analysis

The Shapiro–Wilk and Kolmogorov–Sminov tests showed non-normal distribution of the data collected here; therefore, non-parametric Wilcoxon matched pair test was used to compare paired groups and Spearman’s correlation test was performed to determine associations from changes in variables that occurred after papaya consumption. Statistical analysis was performed using SPSS (version 22.0). *p* < 0.05 was considered significant. Results were presented as mean ± SD.

## Results

### Only NK Cells and a CD69^+^ Subpopulation Were Significantly Different between the Sexes

Comparison between males and females for all parameters combined for the two time points showed significantly lower percentages of total CD3-CD56/16^+^ NK cells in females. Interestingly, a non-CD4 lymphocyte subpopulation with activated features (CD45RA^−^CD69^+^CD25^−^) was significantly higher (10.4 ± 9.4 vs 5.3 ± 2.3, *p* = 0.032, *n* = 32) in females compared to males. As expected, the sex hormones levels were significantly different between males and females (Table 1).

**Table 1:** Mean ± SD values of sex hormones and immune parameters in healthy males and females, combined (all samples) and pre- and post-papaya consumption.

						Pre-papaya vs post-papaya											
	All samples					All subjects			Male			Female			All	M	F
					
	Male	*n*	Female	*n*	M vs F	Pre-	Post-	*n*	Pre-	Post-	*n*	Pre-	Post-	*n*	(Pre- vs Post-)		
**Sex hormone**																	
Estradiol (pmol/l)	98.1 ± 26.5	18	396.9 ± 429.9	18	*P* = 0.000	191.5 ± 147.9	303.5 ± 451.9	18	100.3 ± 23.9	95.8 ± 30.1	9	282.7 ± 165.0	511.1 ± 579.7	9	*p* = 0.107	*p* = 0.594	*p* = 0.036
Progesterone (nmol/l)	0.3 ± 0.0	18	6.4 ± 9.7	18	*p* = 0.010	2.8 ± 6.8	4.0 ± 8.1	18	0.3 ± 0.0	0.3 ± 0.0	9	5.2 ± 9.2	7.61 ± 0.5	9	*p* = 0.039	*p* = 1.000	*p* = 0.039
Testosterone (nmol/l)	7.5 ± 2.5	18	0.8 ± 0.5	18	*p* = 0.000	4.3 ± 4.0	4.0 ± 3.8	18	7.8 ± 2.5	7.2 ± 2.7	9	0.8 ± 0.4	0.8 ± 0.7	9	*p* = 0.248	*p* = 0.129	*p* = 1.000

**Lymphocyte subpopulation**																	
CD3^+^CD4^+^ %	32.9 ± 6.1	30	37.1 ± 8.9	36	*p* = 0.056	34.8 ± 8.3	35.6 ± 7.8	33	32.4 ± 6.1	33.4 ± 6.4	15	36.8 ± 9.4	37.5 ± 8.5	18	*p* = 0.009	*p* = 0.040	*p* = 0.107
CD3^+^CD4^+^ cnt	858.5 ± 264.2	30	980.1 ± 543.2	36	*p* = 0.643	927.6 ± 485.2	922.0 ± 397.6	33	857 ± 254.6	860.0 ± 282.5	15	986.4 ± 617.8	973.7 ± 475.0	18	*p* = 0.688	*p* = 0.910	*p* = 0.647
CD3^+^CD8^+^ %	28.1 ± 9.1	30	28.7 ± 6.7	36	*p* = 0.610	28.4 ± 8.0	28.5 ± 7.7	33	28.3 ± 9.3	28.0 ± 9.1	15	28.5 ± 7.0	29.0 ± 6.6	18	*p* = 0.712	*p* = 0.694	*p* = 0.217
CD3^+^CD8^+^ cnt	748.4 ± 352.0	30	719.0 ± 295.1	36	*p* = 0.872	741.2 ± 345.0	723.6 ± 297.9	33	766.8 ± 372.7	730.1 ± 342.0	15	719.8 ± 329.6	718.2 ± 265.8	18	*p* = 0.761	*p* = 0.551	*p* = 0.327
CD3^−^CD16^+^ 56%	16.8 ± 5.9	30	13.6 ± 5.2	36	*p* = 0.031	15.7 ± 6.0	14.4 ± 5.5	33	17.1 ± 6.0	16.4 ± 6.1	15	14.6 ± 5.8	12.7 ± 4.4	18	*p* = 0.010	*p* = 0.233	*p* = 0.018
CD3^−^CD16^+^56 cnt	465.3 ± 274.9	30	343.5 ± 202.5	36	*p* = 0.053	413.2 ± 245.1	384.5 ± 245.9	33	474.6 ± 264.9	456.1 ± 293.5	15	362.1 ± 221.7	324.8 ± 185.9	18	*p* = 0.183	*p* = 0.650	*p* = 0.157
CD3^−^CD19^+^ %	13.6 ± 3.5	30	14.9 ± 5.2	36	*p* = 0.728	14.1 ± 4.4	14.4 ± 4.7	33	13.7 ± 3.8	13.5 ± 3.4	15	14.5 ± 5.0	15.2 ± 5.5	18	*p* = 0.216	*p* = 0.368	*p* = 0.037
CD3^−^CD19^+^ cnt	362.8 ± 151.7	30	373.1 ± 199.3	36	*p* = 0.880	370.0 ± 194.3	366.8 ± 163.1	33	371.4 ± 153.7	354.2 ± 154.7	15	368.9 ± 227.1	377.4 ± 173.6	18	*p* = 0.531	*p* = 0.460	*p* = 0.149

**Expression of differentiation markers in lymphocyte subpopulations**																	
**CD4^+^ helper T cells**																	
CD4^+^CD45RA^+^	14.3 ± 4.3	16	16.2 ± 6.4	16	*p* = 0.515	15.6 ± 5.4	15.0 ± 5.7	16	14.9 ± 5.1	13.8 ± 3.5	8	16.3 ± 5.8	16.2 ± 7.4	8	*p* = 0.277	*p* = 0.161	*p* = 0.889
CD69^−^CD25^−^	52.5 ± 12.6	16	53.1 ± 12.5	16	*p* = 0.838	52.5 ± 11.8	53.1 ± 13.3	16	51.6 ± 11.9	53.5 ± 14.0	8	53.4 ± 12.3	52.7 ± 13.4	8	*p* = 0.535	*p* = 0.263	*p* = 0.779
CD69^−^CD25^+^	42.7 ± 13.6	16	40.3 ± 13.0	16	*p* = 0.539	41.4 ± 12.5	41.6 ± 14.1	16	43.4 ± 13.2	42.1 ± 14.8	8	39.3 ± 12.4	41.2 ± 14.4	8	*p* = 0.836	*p* = 0.483	*p* = 0.183
CD69^+^CD25^−^	2.8 ± 2.5	16	4.0 ± 3.5	16	*p* = 0.323	3.7 ± 3.5	3.0 ± 2.6	16	3.0 ± 2.8	2.6 ± 2.2	8	4.5 ± 4.1	3.5 ± 3.0	8	*p* = 0.013	*p* = 0.233	*p* = 0.028
CD69^+^CD25^+^	2.0 ± 2.0	16	2.7 ± 2.1	16	*p* = 0.184	2.5 ± 2.1	2.2 ± 2.0	16	2.1 ± 2.1	1.9 ± 2.0	8	2.9 ± 2.2	2.6 ± 2.2	8	*p* = 0.046	*p* = 0.171	*p* = 0.149
CD4^+^CD45RA^−^	13.9 ± 3.0	16	16.3 ± 4.8	16	*p* = 0.184	15.3 ± 4.1	14.9 ± 4.2	16	13.9 ± 2.7	14.0 ± 3.4	8	16.7 ± 4.9	15.9 ± 5.0	8	*p* = 0.778	*p* = 0.866	*p* = 0.735
CD69^−^CD25^−^	46.9 ± 12.2	16	46.3 ± 12.3	16	*p* = 0.590	46.1 ± 11.4	47.0 ± 13.1	16	45.8 ± 11.6	48.0 ± 13.5	8	46.5 ± 11.9	46.1 ± 13.5	8	*p* = 0.501	*p* = 0.326	*p* = 0.779
CD69^−^CD25^+^	51.8 ± 12.3	16	51.6 ± 12.4	16	*p* = 0.780	52.0 ± 11.6	51.4 ± 13.1	16	52.8 ± 11.8	50.8 ± 13.5	8	51.2 ± 12.1	52.0 ± 13.5	8	*p* = 0.605	*p* = 0.327	*p* = 0.575
CD69^+^CD25^−^	0.6 ± 0.3	16	0.9 ± 0.8	16	*p* = 0.239	0.8 ± 0.7	0.6 ± 0.6	16	0.6 ± 0.3	0.5 ± 0.3	8	1.1 ± 0.8	0.8 ± 0.9	8	*p* = 0.007	*p* = 0.066	*p* = 0.041
CD69^+^CD25^+^	0.7 ± 0.3	16	1.2 ± 1.0	16	*p* = 0.305	1.0 ± 0.8	0.9 ± 0.8	16	0.8 ± 0.4	0.7 ± 0.3	8	1.3 ± 1.0	1.1 ± 1.0	8	*p* = 0.045	*p* = 0.121	*p* = 0.180

**CD4^−^ lymphocytes**																	
CD4^−^CD45RA^+^	47.6 ± 5.4	16	43.7 ± 7.3	16	*p* = 0.119	47.5 ± 5.1	43.9 ± 7.6	16	48.2 ± 5.1	47.1 ± 5.9	8	46.8 ± 5.4	40.7 ± 8.1	8	*p* = 0.009	*p* = 0.401	*p* = 0.012
CD69^−^CD25^−^	91.4 ± 7.2	16	90.4 ± 7.0	16	*p* = 0.491	90.2 ± 7.7	91.6 ± 6.3	16	90.7 ± 8.3	92.2 ± 6.4	8	89.7 ± 7.6	91.1 ± 6.7	8	*p* = 0.039	*p* = 0.161	*p* = 0.093
CD69^+^CD25^−^	8.0 ± 7.0	16	9.1 ± 7.1	16	*p* = 0.539	9.3 ± 7.7	7.8 ± 6.4	16	8.8 ± 8.0	7.2 ± 6.3	8	9.8 ± 7.9	8.3 ± 6.8	8	*p* = 0.016	*p* = 0.093	*p* = 0.093
CD4^−^CD45RA^−^	24.1 ± 5.6	16	23.8 ± 10.3	16	*p* = 0.402	21.7 ± 5.7	26.2 ± 9.7	16	23.0 ± 5.6	25.2 ± 5.8	8	20.3 ± 5.8	27.3 ± 12.8	8	*p* = 0.019	*p* = 0.123	*p* = 0.123
CD69^−^CD25^−^	93.5 ± 2.1	16	89.0 ± 9.5	16	*p* = 0.094	91.2 ± 6.2	91.3 ± 8.2	16	93.3 ± 2.0	93.7 ± 2.3	8	89.1 ± 8.2	88.9 ± 11.2	8	*p* = 0.836	*p* = 0.484	*p* = 0.779
CD69^+^CD25^−^	5.3 ± 2.3	16	10.4 ± 9.4	16	*p* = 0.032	7.7 ± 6.4	8.0 ± 8.2	16	5.2 ± 2.4	5.5 ± 2.4	8	10.3 ± 8.2	10.6 ± 11.1	8	*p* = 0.918	*p* = 0.889	*p* = 1.000

**Expression of activation markers in cytotoxic lymphocytes**																	
CD8^+^ cytotoxic T cells																	
CD3^+^CD8^+^IFN^+^	6.0 ± 4.8	16	7.1 ± 5.7	16	*p* = 0.468	7.0 ± 5.4	6.1 ± 5.0	16	6.7 ± 5.7	5.3 ± 3.8	8	7.2 ± 5.5	7.0 ± 6.2	8	*p* = 0.427	*p* = 0.779	*p* = 0.398
CD3^+^CD8^+^IL-12R^+^	4.6 ± 1.7	16	5.4 ± 2.2	16	*p* = 0.590	5.0 ± 2.1	5.0 ± 2.0	16	4.7 ± 2.0	4.6 ± 1.5	8	5.2 ± 2.3	5.5 ± 2.3	8	*p* = 0.660	*p* = 0.944	*p* = 0.441
CD3^+^CD8^+^IL-15R^+^	8.4 ± 4.5	18	9.6 ± 5.3	16	*p* = 0.422	9.5 ± 4.9	8.4 ± 4.9	17	8.6 ± 4.5	8.2 ± 4.7	9	10.5 ± 5.4	8.7 ± 5.4	8	*p* = 0.218	*p* = 0.678	*p* = 0.182
CD3^+^CD8^+^IL-21R^+^	7.2 ± 2.8	16	8.6 ± 3.6	16	*p* = 0.341	8.1 ± 3.5	7.7 ± 3.0	16	7.1 ± 3.5	7.2 ± 2.1	8	9.0 ± 3.5	8.2 ± 3.8	8	*p* = 0.642	*p* = 0.575	*p* = 0.208
CD3^+^CD8^+^CD107a^+^	5.8 ± 2.3	18	5.3 ± 1.8	16	*p* = 0.851	5.7 ± 2.0	5.5 ± 2.2	18	6.0 ± 2.1	5.6 ± 2.7	9	5.3 ± 2.0	5.3 ± 1.8	9	*p* = 1.000	*p* = 0.767	*p* = 0.799

**Natural killer cells**																	
CD3^−^CD56^+^IFN^+^	21.1 ± 2.1	14	23.1 ± 15.7	14	*p* = 0.946	21.9 ± 14.5	22.2 ± 13.5	14	22.6 ± 13.4	19.5 ± 11.5	7	21.3 ± 16.7	24.9 ± 15.7	7	*p* = 0.683	*p* = 0.176	*p* = 0.612
CD3^−^CD56^+^IL12R^+^	11.2 ± 5.3	16	13.2 ± 6.5	16	*p* = 0.838	11.9 ± 5.7	12.5 ± 6.3	16	11.0 ± 5.3	11.5 ± 5.7	8	12.8 ± 6.2	13.5 ± 7.2	8	*p* = 0.642	*p* = 0.889	*p* = 0.674
CD3^−^CD56^+^IL15R^+^	17.9 ± 11.5	18	20.0 ± 9.9	18	*p* = 0.355	20.3 ± 12.9	17.6 ± 7.7	18	18.4 ± 14.8	17.3 ± 7.6	9	22.1 ± 11.3	17.8 ± 8.3	9	*p* = 0.356	*p* = 0.889	*p* = 0.214
CD3^−^CD56^+^IL-21R^+^	17.8 ± 6.9	16	21.7 ± 10.2	16	*p* = 0.381	20.1 ± 8.5	19.3 ± 9.4	16	16.9 ± 5.8	18.6 ± 8.2	8	23.4 ± 9.8	20.0 ± 11.0	8	*p* = 0.679	*p* = 0.484	*p* = 0.327
CD3^−^CD56^+^CD107a^+^	15.7 ± 5.9	18	16.1 ± 6.9	16	*p* = 0.746	15.1 ± 6.5	16.7 ± 6.2	17	14.7 ± 4.8	16.8 ± 7.0	9	15.6 ± 8.4	16.5 ± 5.7	8	*p* = 0.044	*p* = 0.038	*p* = 0.397

*M, male; F, female; n, number of samples*.

*Statistical significance achieved where p < 0.05*.

### 17β-Estradiol and Progesterone Levels Were Significantly Increased in Females after Papaya Consumption

**Figure 1:**
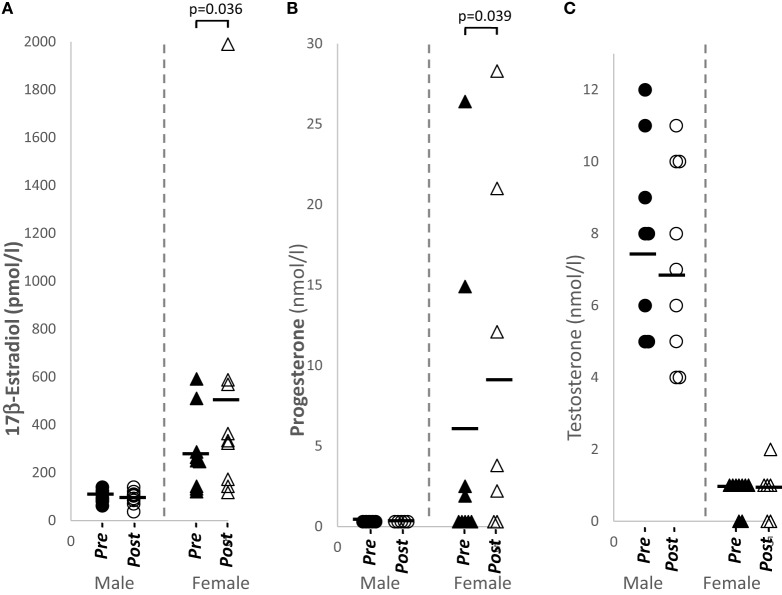
Distribution of sex hormone levels in healthy human males (*N* = 9) and females (*N* = 9), pre- and post-papaya consumption. **(A)** 17β-estradiol, **(B)** progesterone, and **(C)** testosterone. *Statistical significance achieved where *p* < 0.05.

Plasma sex hormone levels of 17β-estradiol (*p* = 0.036, *n* = 9) and progesterone (*p* = 0.039, *n* = 9) were significantly increased in females after papaya consumption (Figure 1; Table 1). Even though the experiment was designed to be carried out during the follicular phase of the female menstrual cycle, two subjects showed luteal phase levels for progesterone. These samples were not excluded as this study analyzed pre- and post-levels. None of the hormones tested demonstrated significant change in males after *C. papaya* consumption.

### Total NK Cell Percentages Were Significantly Reduced in Females while Total CD4^+^ T Cell and Total B Cell Were Significantly Increased in Males and Females, Respectively, after Papaya Consumption

**Figure 2:**
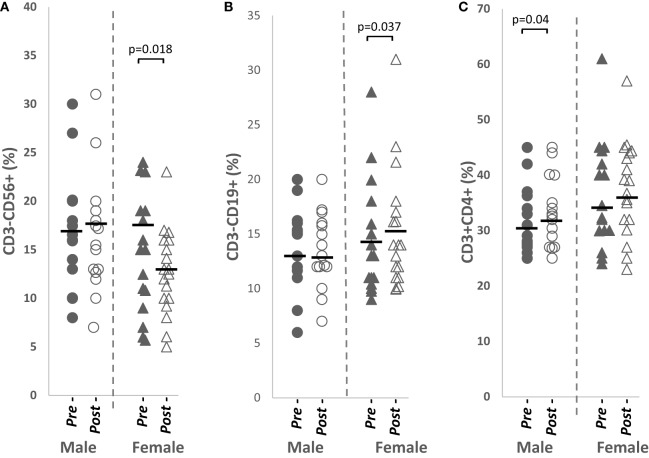
Distribution of **(A)** total CD3^−^CD56^+^ NK cells, **(B)** total CD3^−^CD19^+^ B cells, and **(C)** total CD3^+^CD4^+^ helper T cells percentages in healthy males (*n* = 15) and females (*n* = 18) pre- and post-papaya consumption. *Statistical significance achieved where *p* < 0.05.

Total NK cells from peripheral blood were significantly downregulated (*p* = 0.018, *n* = 18), while total B cell percentages were significantly increased (*p* = 0.037, *n* = 18) in females after papaya consumption (Figure 2). Total CD4^+^ T cells was significantly increased (*p* = 0.040, *n* = 15) in males (Table 1).

A negative association was detected between change in 17β-estradiol levels and change in NK cell percentages in females (*R* = −0.586, *p* = 0.097, *n* = 9) suggesting increased 17β-estradiol may play a role in downregulating NK cells. No significant correlation was observed between sex hormone and total B cells in females or total CD4^+^ T cells in males.

### CD69^+^ T Cells Were Significantly Reduced after Papaya Consumption

**Figure 3:**
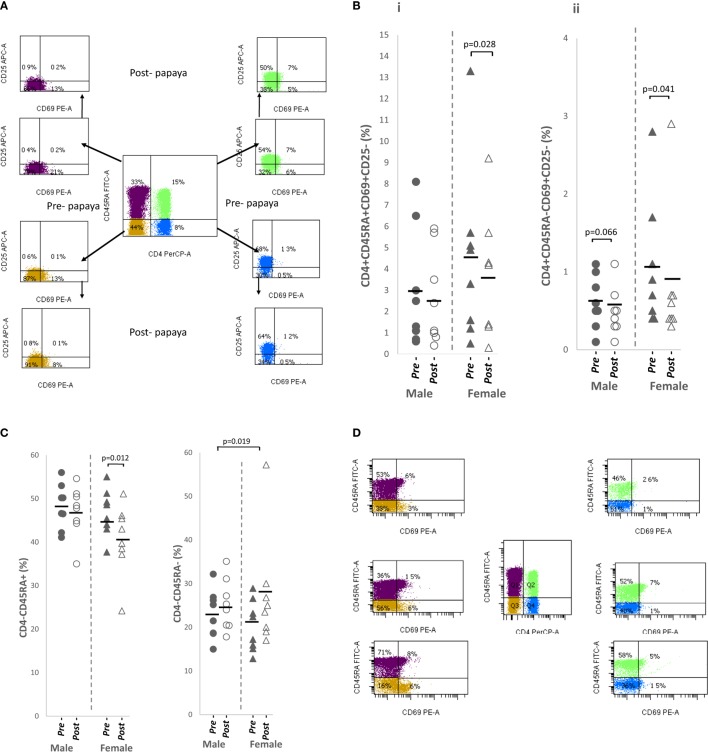
**(A)** Flow cytometry gating strategy for analysis of differentiation markers expressed on CD4^+^ T cells/non-CD4^+^ lymphocytes in lysed whole blood. Initial gating was on lymphocytes with low SSC/low FSC. Distribution of **(B)** CD69^+^ in CD4^+^ T cell subpopulations i. naïve, CD4^+^CD45RA^+^CD69^+^CD25^−^ and ii. activated, CD4^+^CD45RA^−^CD69^+^CD25^−^, and **(C)** non-CD4^+^ lymphocytes i. total naïve CD4^+^CD45RA^+^ and ii. total activated CD4^+^CD45RA^−^ in healthy males (*n* = 8) and females (*n* = 8) pre- and post-papaya consumption. **(D)** Representative flow cytometry plots showing CD69^+^ fractions in naïve, CD45RA^+^ and activated CD45RA^−^ CD4^+^ T cells and non-CD4^+^ lymphocytes. *Statistical significance achieved where *p* < 0.05.

Three differentiation markers (CD45RA, CD69, and CD25) were selected from literature based on their use as naïve and activated/effector markers (Figure 3A). Table 1 shows comparable percentages of naïve, CD4^+^CD45RA^+^ and non-naïve, CD4^+^CD45RA^−^ T helper cells. Among naïve cells, the double negative, CD69^−^CD25^−^ subpopulation made up a major proportion followed by CD69^−^CD25^+^ single positive cells. Within non-naive cells, mean percentage of the double negative subpopulation was lower while CD69^−^CD25^+^ cells were higher (Table 1).

CD69 expression (CD25^+^/CD25^−^) was observed on only a small fraction (2.0–4.0%) of naïve cells and was lower (0.6–1.2%) among non-naïve T helper cells (Table 1). The non-naïve (CD45RA^−^) component consisted of activated, effector, memory as well as regulatory cells, thus these cells may also be referred as activated cells here.

A relatively large mean percentage of CD25^+^ cells was observed in the naïve component (40–45%) and was higher in activated CD4^+^ T cells (Table 1).

All CD69-expressing T cells, either single CD69^+^CD25^−^ or double positive CD69^+^CD25^+^ were in general, significantly downregulated in naïve and activated subpopulations after papaya consumption (Figure 3B; Table 1). Correlation analysis with sex hormones revealed negative associations [*R* ≤(−)0.591] with progesterone in females and testosterone in males but these associations were insignificant (*p* ≥ 0.116).

Correlation analysis between sex hormones and CD25-expressing cells, however, revealed significant strong negative associations between changes in testosterone levels and percentages of CD25-expressing T helper cells, in a naïve CD4^+^CD45RA^+^CD69^−^CD25^+^ (*R* = −0.899, *p* = 0.002, *n* = 8) and the activated subpopulations, CD4^+^CD45RA^−^CD69^−^CD25^+^ (*R* = −0.894, *p* = 0.003, *n* = 8) and CD4^+^CD45RA^−^CD69^+^CD25^+^ (*R* = −0.852, *p* = 0.007, *n* = 8), in males following papaya consumption (Figure 2). A “mirror image” significant strong positive correlations were observed between testosterone with double-negative (CD69^−^CD25^−^) naïve (*R* = 0.894, *p* = 0.003, *n* = 8) and activated (*R* = 0.899, *p* = 0.002, *n* = 8) CD4^+^ T cells (Table 1).

Progesterone also had an apparent suppressive effect on CD25^+^ cells in females, as negative correlations were observed with single positive, naïve CD45RA^+^CD69^−^CD25^+^ (*R* = −0.524, *p* = 0.183, *n* = 8) and activated CD45RA^−^CD69^−^CD25^+^ (*R* = −0.600, *p* = 0.116, *n* = 8) T helper cells, following papaya consumption. The relation, however, was not as strong as testosterone in males.

### Total Naïve Non-CD4^+^ Lymphocytes Were Significantly Reduced while Total Activated Non-CD4^+^ Lymphocytes Significantly Increased after Papaya Consumption

Non-CD4^+^ (CD4^−^) lymphocytes were a mixed population consisting of CD8 T cells, NK, B and NKT subsets. CD25 positivity was very low among these cells, <1% (data not shown) and excluded from further analysis.

The majority of non-CD4^+^ lymphocytes, were double negative (CD69^−^CD25^−^). Compared to CD4^+^ lymphocytes where expression of CD69 was found on 0.6–4.0%, a larger population of CD69^+^ cells was observed among the non-CD4^+^ lymphocytes forming average percentages of 8.0–9.1% in CD45RA^+^ and 5.3–10.4% in CD45RA^−^ lymphocytes (Table 1).

Total naïve non-CD4^+^ lymphocytes were significantly reduced while the activated populations were significantly increased after papaya consumption (Table 1). Distribution of pre- and post-papaya consumption percentages are shown in Figure 3C. Among naïve cells, the CD69^+^CD25^−^ subpopulations were significantly reduced while the corresponding double negative (CD69^−^CD25^−^) subpopulations were significantly increased (Table 1). Within the activated population, changes were not significant as modulations were more heterogeneous between individual subjects which resulted in less obvious total effect. Interestingly, CD69^+^CD25^−^ subpopulations were larger in females, particularly the significantly higher activated CD4^−^CD45RA^−^CD69^+^CD25^−^ subpopulation (Table 1). Negative correlations were observed between naïve and activated CD69^+^ subpopulations and testosterone in males (*R* = −0.728, *p* = 0.041, *n* = 8 and *R* = −0.664, *p* = 0.073, *n* = 8, respectively) as well as progesterone in females (*R* = −0.434, *p* = 0.282, *n* = 8 and *R* = −0.668, *p* = 0.070. *n* = 8, respectively), though the majority of these correlations were not significant.

Interesting also to note, CD69 expression was associated with two divergent levels of CD45RA expression, i.e., CD45RA^hi^CD69^+^ and CD45RA^−^CD69^+^ (Figure 3D). CD69 was not expressed on cells with intermediate levels of CD45RA. These may be useful in differentiating circulating regulatory cells/early activated cells before cell division, and memory/effector cells/migrating Tregs, respectively.

### No Significant Change in CD8^+^ T Cell Subsets Expressing Effector Markers after Papaya Consumption

The distinctly increased activated non-CD4^+^ cells after papaya consumption prompted a closer examination of this population, consisting of CD8^+^ T cells, B cells, NK cells, or NKT cells. We opted for the cytotoxic component for further analysis and selected several activation markers associated with these cells. Effector markers analyzed were IFN-γ, IL-12R2β, IL-15Rα, IL-21R, and degranulation marker, CD107a.

No significant changes were observed in CD8^+^ T cells expressing any of these markers (Table 1).

### Significantly Increased CD107a^+^ NK Cells after Papaya Consumption

The same effector markers were analyzed on NK cells (CD3^−^CD56^+^). By comparison, these markers were expressed on a larger percentage of NK cells compared to CD8^+^ T cells (Table 1).

**Figure 4:**
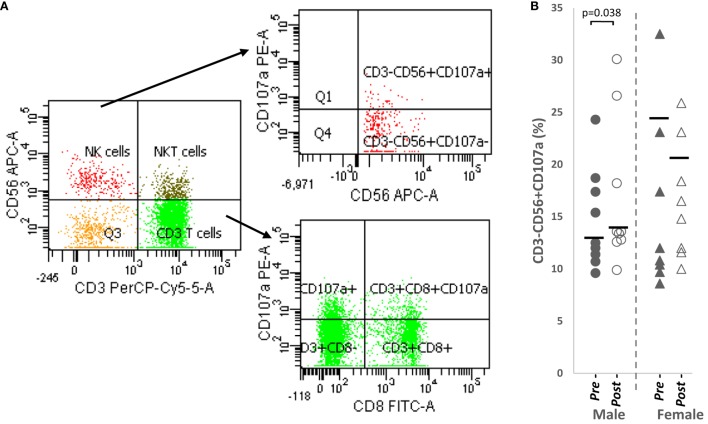
**(A)** Flow cytometry gating strategy to analyze degranulation marker, CD107a on CD3^+^CD8^+^ cytotoxic T cells and CD3^−^CD56^+^ NK cells. Initial gating was on lymphocytes with low SSC/low FSC **(B)** distribution of CD107a NK cell percentages in healthy male (*n* = 9) and females (*n* = 8) pre- and post-papaya consumption. *Statistical significance achieved where *p* < 0.05.

Overall, a significant upregulation of CD107a^+^ NK cells was observed after papaya consumption (Figure 4B; Table 1) particularly in males. Representative flow cytometry plots demonstrating gating strategy to detect CD107a expression on cytotoxic cells are shown in Figure 4A.

Correlation analysis revealed CD107a^+^ NK cells no strong correlation with testosterone levels in males (*R* = 0.520, *p* = 0.151, *n* = 9) but demonstrated strong positive association (*R* = 0.771, *p* = 0.025, *n* = 8) with progesterone in females. Thus, while papaya generally induced NK cell degranulation, in females, this occurred only in increased progesterone.

## Discussion

In this study, the feasibility of detecting endo-immunomodulation by dietary intake of *Carica papaya* fruit was explored in an oral sensory receptive model involving a small population of apparently healthy individual. This preliminary, 2-day short-term exposure revealed interesting results.

Exogenous supplementation from plant-based hormones may affect outcomes in the study as fruits and vegetables contain a myriad of phytochemicals including phytoestrogens. However, a study on premenopausal women given isoflavone-rich diets was not shown to affect serum estradiol or progesterone concentrations (19).

We observed significantly increased 17β-estradiol (E2) and progesterone (P4) in females after papaya consumption. Researches on effects of whole fruits on sex hormones in premenopausal women are limited. In the BioCycle Study on healthy premenopausal women, increased intake of citrus fruit juice did not alter estradiol levels but increased progesterone levels. No significant changes were observed, however, with increased intake of non-citrus fruit juice (20). In healthy postmenopausal women, whole grapefruit significantly increased estrone-3-sulfate (E1S) while fresh juice, bottled juice, and soda intake significantly lowered estradiol (E2) (21). Pomegranate juice reduced estrone (E1) and testosterone in normal weight postmenopausal women (22). Thus, our results support evidence of potential sex hormone alterations from intake of fruits in women.

Estrogen receptor (ER) and progesterone receptors are expressed on various lymphocytes [reviewed in Ref. (23)]. Total NK cell percentages significantly reduced in females consuming papaya in this study was shown to have a negative association with 17β-estradiol. Several conditions have shown that NK cell counts are decreased by estrogen. Peripheral NK cells are reduced during pregnancy (24). *In vivo* application of ethinyl estradiol in transsexual male resulted in significant decrease in percentages of NK cells (25). These studies support our observation and suggest increased estradiol levels in females may have contributed to reduced percentage of NK cells in females.

Other researchers found estrogen replacement therapy in postmenopausal women especially increased B-lymphocyte numbers and decreased pro-inflammatory cytokine production (26, 27). ERβ is upregulated in B cells (23). The increased 17β-estradiol levels and total B cells seen here may be similar responses as reported.

Differentiation markers such as CD45RA, CD69, and CD25 are extensively used in literature but comparison across lymphocyte subpopulations in the system is few. Human naive and memory T cells have been identified by the reciprocal expression of the CD45RA and CD45RO isoforms. The peripheral blood reportedly, contains a comparable proportion of CD45RO^+^ and CD45RA^+^ subsets (28), as was similarly observed here.

CD69 and CD25 are regarded as early and late activation markers, respectively, as an early peak in expression (24 h) of CD69 and a later (48 h) peak in expression of CD25 after *in vitro* phytohemagglutination (PHA) stimulation of CD45RA/CD45RO CD4^+^ T cell subsets were observed (29). Since then, CD69 expression has been induced *in vitro* on cells of most hematopoietic lineages, including T and B lymphocytes, NK cells, murine macrophages, neutrophils, and eosinophils, while it is constitutively expressed on human monocytes, platelets, and epidermal Langerhans cells (30).

Even though, the specific ligand for CD69 has not been identified and the role of CD69 is currently intensively investigated. CD69-expressing T cells, CD4^+^CD69^+^CD25^−^ has been proposed as a novel regulatory cell type defined by TGF-β1 activity (31). The suppressive function of Tregs is dependent on CD69 expression, which forms approximately fifty percent of total CD4^+^CD25^+^FoxP3^+^ Tregs in thymus and secondary lymphoid organs (32). Another major function, however, is involvement in immune cell migration. CD69 is expressed at high levels on approximately 10–15% of thymocytes and play a role in selection and maturation processes in the thymus (33). This may explain the small fraction of CD69^+^ cells (CD25±) found among naïve (CD45RA^+^) T helper cells in this study. A study on tissues acquired from deceased organ donors, however, showed CD45RA^+^ T cells were predominantly CD69 negative, including 100% of naive T cells in blood (28).

Brenchley et al. (34) examined expressions of differentiation markers in activated, proliferated and effector T cells after *in vitro* stimulation. CD25 and CD45RO become expressed on activated cells prior to cell division and maintained thereafter. CD45RA and CD69 were both down-modulated in a cell division dependent fashion, i.e., mitotic dilution, after stimulation. An expansion period for activated naïve T-cells may be more important before acquisition of effector function, after which some differentiate into resting, antigen-experienced T-cells. These results correspond with observations here where CD69 expression was lower in the non-naïve/activated population. This also suggested that activated cells (CD69^+^) were in the “naïve” populations. Furthermore, migratory role of CD69 has also been demonstrated following activation. In inflamed lymph nodes, CD69 is upregulated on lymphocytes, which control its movements within (35). A small fraction (1–20%) of circulating memory (CD45RO^+^) cells express CD69 (28). CD69 mediates homing and retention of CD4^+^ T memory cells in the bone marrow (36).

Expression of CD25 has typically been associated with activated cells. However, we observed a large fraction of CD25^+^ cells (40–45%) among naïve T helper cells. Using an extensive number of activation, differentiation, and exhaustion markers combined with microarray analysis, Pekalski et al. (37) confirmed existence of a subset of naive CD4^+^CD45RA^+^ T cells that express CD25. The percentage of CD4^+^CD25^+^ naive T cells was strongly associated with increasing age and were also detected in cord blood, indicating that acquisition of CD25 expression by naive CD4 T cells begins prior to birth (25). CD25 induction in naïve cells occurs through TCR signaling which, however, are not strong enough to lead to T cell activation and loss or acquisition of markers characterizing effector or memory cells but is important for expansion in the periphery of a naïve TCR repertoire particularly after the period of thymic involution. These cells respond faster and better to low dose IL-2 compared to their CD25^−^ counterpart (37). CD25 is also highly expressed on regulatory T cells. It is now apparent that the naive CD45RA^+^ subpopulation of CD4^+^CD25^hi^FoxP3^lo^ T cells in blood is the most suitable target population for *in vitro* expansion of regulatory T cells (38). These resting Treg cells are induced into activated Tregs to become CD45RA^−^FoxP3^hi^ cells, a population also observed in peripheral blood of healthy individuals (39).

CD25^+^ percentages are higher in non-naïve T helper cells as was seen here. Resting memory T-cells may be CD25^−^, i.e., late differentiated cells that respond to antigens associated with chronic immune responses. The majority however, are CD25(INT) memory T cells that respond to antigens associated with recall responses, produce a greater array of cytokines, and are less dependent on co-stimulation for effector responses due to their expression of CD25 (40).

Many studies have shown levels of CD4^+^ T cells are lower in males compared to females [reviewed in Ref. (23)] as was also observed here. We detected strong negative associations between testosterone and CD25-expressing T cells. These were consistent with another report showing suppressive effect of testosterone on CD25^+^CD45RA^+^ and CD25^+^CD45RO^+^ T cells (41). Medical castration reduced testosterone levels and increased CD4^+^CD25^+^ cells (42). Thus, testosterone appeared to target the CD25^+^ marker. The action of testosterone on the CD25^+^ cells may be to induce cell death as testosterone is shown to induce apoptosis in T cells (43). We also observed decreased percentages of the major populations of CD25^+^ cells (CD4^+^CD45RA^+^CD69^−^CD25^+^/CD4^+^CD45RA^+^CD69^−^CD25^+^) in males (but not in females) as the majority of male subjects maintained/increased testosterone levels after papaya consumption.

In reverse, CD25^−^ cells, both naïve and activated were increased in males (but not females) after papaya consumption. This may be a homeostatic response [discussed in Ref. (44)] in an effort to return T cells to normal levels by inducing proliferation of naïve cells (CD4^+^CD45^+^CD69^−^CD25^−^), which are dominant and robust having indefinite life span. In the presence of androgen, CD4 T cell differentiation inhibition was also demonstrated by significantly reduced levels of Tbet and IFN-γ (45) possibly mediated through upregulation of CD4^+^CD25^+^Foxp3^+^ regulatory T cells (46). In an earlier study, we also demonstrated increased regulatory T cells after papaya consumption (47). The accumulative effect may have been to increase slightly the percentage of CD4^+^ T cells in males, observed here.

The low expression of CD25 on non-CD4^+^ lymphocytes is consistent with other reports; immature B and certain NKT subsets may express low levels of CD25. Mature B cells, NK cells, and NKT are absent for CD25 (48). The alpha (CD25) chain is one of three subunits that make up the IL-2 receptor, the other two being beta (CD122), and gamma (gammac) chains. CD8^+^ T cells preferentially express CD122 and naturally occurring CD8^+^CD122^+^ T cells maintain T cell homeostasis as well as Treg function. Murine CD8^+^CD122^+^ Tregs carry CD122 or IL-2Rβ, but not CD25, while CD4^+^CD25^+^ Tregs do not express CD122, although both subsets of Tregs are CD44^high^, CD62L^high^ and mostly CD127-negative [reviewed in Ref. (49)].

Consumption of papaya in general induced a suppressive effect on CD69^+^ cells, particularly CD4^+^ T helper cells as well as the naïve non-CD4^+^ lymphocytes. The potential of fruits to inhibit CD69 expression has been shown in the *in vitro* administration of auraptene, a citrus fruit-derived coumarin (50) and cactus pear fruit extract (51) on activated lymphocytes.

However, this effect was not similarly observed in activated non-CD4^+^ lymphocytes. Individual responses were heterogeneous and mean percentage was, in reverse, slightly increased after papaya consumption. Negative correlations were generally observed between CD69^+^ subpopulations with testosterone in males and progesterone in females. In fact, the negative correlation with this activated non-CD4^+^ lymphocyte was the strongest in females. The selective nature of progesterone is in concordance with reported evidence of progesterone suppression of uterine natural killer (NK) cells in human and spleen cells in mice expressing CD69 (52). In females, this population of cells appeared to be responsive to the effect of progesterone and resulted in reduced percentages in subjects increased for progesterone. Since not all subjects increased progesterone levels, overall effect of suppression was not significant. More importantly, in subjects with low levels of progesterone, percentages of these cells increased and were not affected by the presumed suppressive effect of papaya. In females, non-naïve CD69^+^non-CD4^+^ lymphocytes formed a significantly larger population compared to males. This may also be due to the majority of females being in the follicular phase of the menstrual cycle with relatively lower levels of progesterone. We were unable to locate literature studying effect of testosterone on CD69^+^ lymphocytes.

The significantly increased NK cell degranulation (CD107a^+^) in males after papaya consumption appeared to be unaffected by sex hormone changes. Other studies strengthen this observation. NK cell activity of peripheral mononuclear cells against target K562 cells measured by the 51Cr release assay did not differ between patients with idiopathic hypogonadotropic hypogonadism (with significantly lower mean plasma testosterone) and healthy adults. Most importantly, this activity did not change during hormonal treatment, which normalized plasma testosterone levels in the patients (53). NK cells from normal donors exhibiting K562 lysis are shown to be CD56^+^CD69^−^. CD56^+^CD69^+^ cells did not significantly increase cytotoxicity even though PMA stimulation increases CD69 expression on NK cells (54). Thus, CD107a NK cells may be CD69 negative. In an earlier study, we also observed significantly increased percentages of NK cells in males (but not females) after *in vitro* PHA activation. The lower response in females did not appear to involve CD69^+^ cells, indirectly confirming no sex hormone effects in the *in vitro* study (55).

In females, increased NK degranulation activity was only observed when progesterone levels were also increased in subjects after papaya consumption. *In vitro*, no effect of progesterone on NK activity was demonstrated but women on oral contraceptive and fertile females in the luteal phase of the cycle have lower NK cell activity than males or post-menopausal women. During the follicular phase, these differences were not apparent. However, the effect may be either from estrogen or progesterone [reviewed in Ref. (56)]. Our results differ from reported evidence, as we observed progesterone may have stimulatory effect on NK activity. Thus, this remains controversial but the action of hormones may be dependent on status of cell activation.

Fruit extracts have been shown to modulate the immune system significantly even within a day of treatment (11, 13). Nevertheless, studies with long term treatments, e.g., of 33 days (12) to 70 days (10) also provide similar evidence of immunomodulatory responses. Thus, regular supplementation may continually induce an immune-related change. However, whether this is a desired change will be dependent on the purported outcome.

The inability to elicit similar sex hormonal changes in all subjects resulting in heterogeneous responses may be due to individual variability, insufficient stimulation with 2 days exposure or observations were just random changes to the physiological environment. However, the inclusion of the sex hormone markers in this study has clarified many dimorphism seen in immune responses that would not have been otherwise understood.

## Conclusion

The vast knowledge available on the immune system allowed us to better interpret complex changes from normal exposures. The short-term papaya consumption experiment revealed sexual dimorphic changes in the immune system. Both stimulatory and suppressive effects were observed in lymphocyte subsets of healthy individuals after papaya consumption. Stimulation of CD4^+^ T cell percentages and NK cell activity in males suggest a beneficial potential from papaya consumption in this subset of individuals. Increased B cell percentages and reduced percentages of NK cells are characteristics of the female immune profile. It is not clear if “exacerbation” of these situations with papaya consumption may not be advantageous. Similarly, decreased naïve non-CD4^+^ lymphocytes seen in females may not be desirable. This study also revealed endocrine–immune system interactions, in particular, the possible suppressive effect of testosterone on CD25. Furthermore, low progesterone levels, e.g., during the follicular phase appeared to promote activated CD69^+^ non-CD4^+^ lymphocytes but led to non-responsiveness in NK degranulation inducible by external factors such as papaya consumption, as observed here.

Due to a spectrum in expression of these markers across normal individuals, an overlap of phenotypes did occur between sexes, thus no strict “sex-labeled” boundaries existed. However, sex-biased responses were still distinguishable and sex hormone levels were able to provide a guide. The ability to measure immune response *in vivo* fulfills an important facet in the overall evaluation of immune health. The limitations of this study were the short supplementation period and the small number of samples analyzed. A larger number of subjects and a longer period of supplementation will be required to confirm these results.

## Ethics Statement

This study was approved by Medical Research Ethics Committee, Faculty of Medicine and Health Sciences, Universiti Putra Malaysia. All procedures complied with the principles of the Declaration of Helsinki. Informed consents were obtained.

## Author Contributions

MA, ZS, RJ, and WK contributed to the conception and design of the study. NJ, CY, and MA contributed to acquisition of data, analysis, and interpretation of data. MA and NJ drafted the article and revised it critically for important intellectual content. All authors approved the final the version to be submitted.

## Conflict of Interest Statement

The authors declare that the research was conducted in the absence of any commercial or financial relationships that could be construed as a potential conflict of interest.
